# Can we mimic 3D printing of low molecular weight gels using a rheometer? – a characterisation toolkit for extrusion printed gels†

**DOI:** 10.1039/d4fd00185k

**Published:** 2024-12-09

**Authors:** Rebecca E. Ginesi, James Doutch, Emily R. Draper

**Affiliations:** a School of Chemistry, University of Glasgow Glasgow G12 8QQ UK Emily.Draper@glasgow.ac.uk; b Centre for the Cellular Microenvironment, School of Molecular Biosciences, The Advanced Research Centre, University of Glasgow 11 Chapel Lane Glasgow G11 6EW UK; c ISIS, Rutherford Appleton Laboratory Oxfordshire OX11 0QX UK

## Abstract

The 3D printing of hydrogels from low molecular weight gelators (LMWGs) continues to attract notable interest, with many potential applications. One of the main issues with 3D printing is the difficulty characterising these gels after printing. Currently, the understanding of whether these bulk rheological properties are maintained upon printing is limited. To address this, we have developed a series of rheological and scattering methods to characterise these materials before, during, and after printing. We have used rheology and small-angle neutron scattering (SANS) to gain a deeper understanding of the impact printing has on the bulk properties of the hydrogels. We have determined that printing impacts the resulting gel fibril structure, which consequently changes the stiffness and strength of the gel. We hope that through this work, we have provided advances to the field of 3D printing of LMWGs, as well as showing the versatility of this fabrication technique to create gels with different properties.

## Abstract 2

We have developed a series of rheological and scattering methods to characterise hydrogels 3D printed from low molecular weight gelators, before, during, and after printing.

## Graphical abstract

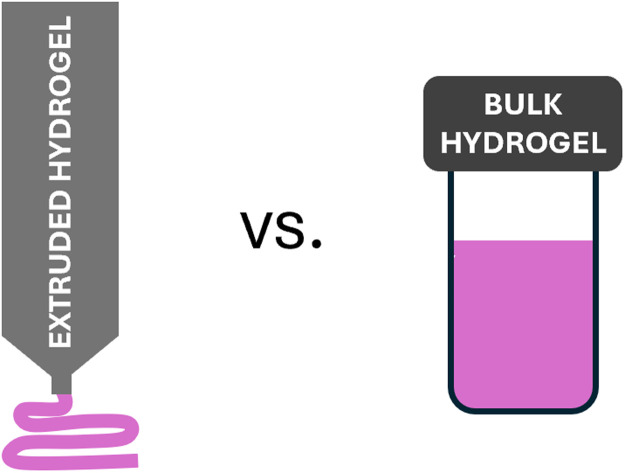

## Introduction

Printable hydrogels are gaining interest in a wide variety of fields, from precision medicine to optoelectronics.^1–3^ During 3D printing, a material (also sometimes referred to as an ink) is continuously deposited in a layer-by-layer manner.^4^ As such, 3D printing allows for the preparation of more complex shapes and fine structures and is more precise and less time-consuming than using moulds.^2,3,5^ Extrusion printing of polymer- and peptide-based hydrogels has been well reported.^6,7^ Typically, these materials are expelled as pre-gel solutions that undergo gelation post-printing *via* a gelation trigger, such as exposure to cross-linking agents,^8^ temperature change,^9^ or UV photocuring.^10^ However, polymer gels can be limited by their irreversibility and lack of shear thinning properties. Furthermore, some cross-linking agents used to trigger gelation can be toxic,^11^ which limits their use in biomaterials. 3D printing of low molecular weight gelators (LMWGs) is scarcely described in the literature, with many LMWGs requiring an additive to improve their printability. Unlike chemical gels, some supramolecular gels can be formed before printing,^2,12^ allowing the properties of the final printed (extruded) material to be potentially pre-defined.^2,13^ When gelation is triggered after printing, it can be difficult to predict or pre-determine the resulting properties. As the purpose of printing is typically to print these gels with precise shapes, properties, and networks for the chosen application, it is more favourable to pre-form and subsequently extrude the gel. However, this requires that the material be printable and that its properties do not change (or at least recover) upon printing.

For hydrogels to be 3D printed (or extrusion printed), it is crucial to understand the printability of the precursor gel.^14^ The printability is related to the behaviour of the material whilst being sheared during extrusion (known as extrudability) and its performance and stability post-printing (denoted by shape fidelity). The rheological properties of the hydrogel are the physicochemical parameters that have the greatest influence on its behaviour throughout the 3D printing process.^15,16^ The material viscosity and shear-thinning properties will determine how easily it flows through the syringe nozzle and impact its ability to maintain its shape after extrusion. The thixotropy of a gel (*i.e.* the steady decrease in viscosity over time for a constant applied shear stress, followed by a gradual recovery when this stress is removed) is another crucial parameter that determines the suitability of hydrogels for printing. In extrusion printing, shear-thinning relates to the ease of extrusion and the shape preservation of the gel as it is printed. When the material moves through the syringe nozzle, its viscosity is reduced due to the large increase in applied shear causing shear-thinning. Thus, a quick recovery time is a desirable property of the hydrogel.^14^

Currently, most reported examples of hydrogels suitable for printing have been discovered through serendipity.^2^ Therefore, the link between the microstructure of the gel network and its printability is poorly understood. In the literature, the suitability of a hydrogel for 3D printing is commonly assessed by characterising its mechanical properties before printing, with little, if any, rheological measurements done post-printing and almost never during printing.^17^ It is assumed that the mechanical properties of the printed gel are unaffected by the printing process, which seems unlikely due to the processes involved. There are many examples of LMWGs being process-dependent (for example, gel-to-gel transitions),^18–21^ and so this is an unusual assumption to make. This lack of analysis is likely due to the difficulty in carrying out such characterisations. However, such information is crucial to determine the types of applications these materials are suitable for.

Here, we have developed a series of rheological methods to address these challenges. Crucially, we utilise both rheology and small-angle neutron scattering (SANS) to characterise the properties of amino acid-appended perylene bisimides/polymer-based hydrogels before, during, and after printing to understand the impact of the printing process and the effect on the bulk properties. We sought to see if a shear recovery test commonly performed on a rheometer (and often used to assume ‘printability’) can be a good proxy for extruded gels through a needle. Therefore, we performed *in situ* RheoSANS before, during, and after shearing to collect structural and mechanical information on our gels. This information was then compared to data collected from printed gels at the same shear rate.

## Results and discussion

**Fig. 1:**
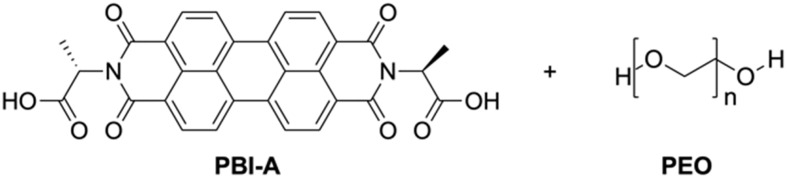
Chemical structures of PBI-A and PEO used in this study.

We focus here on PBI-A (Fig. 1), a well-studied LMWG within our group,^22–25^ with interesting photoelectric behaviour due to the formation of a radical anion upon exposure to UV light,^22,26–28^ and is very much of interest in a variety of applications, especially in the gel state with temporal control being of high interest. However, PBI hydrogels are typically soft, which may lead to fragile architectures upon printing.^29,30^ Many groups have shown that using polymer additives can modulate the properties of supramolecular hydrogels, such as making them stronger.^31–33^ Therefore, a non-gelling polymer additive was added to create PBI-A/polymer blends. The polymer must not interfere with the existing properties of the PBI gel (optical, conductivity, *etc.*) and not alter the mechanical properties in a way that makes them unprintable. PEO (*M*_w_ = 500 000) was chosen for the blends due to these requirements, but other polymers were tested (Table S1, ESI†). The blend composition was altered by adjusting the percentage volume of the PBI and polymer (which will be quoted here as ratios of PBI-A/PEO).

PBIs act as surfactants, meaning their aggregation state is likely dependent on concentration.^25^ As such, the final concentration of PBI-A and PEO in each blend was maintained at 5 mg mL^−1^. Therefore, any differences in the blends would be due to the volumes of the PBI-A and polymer in each blend, and the aggregates formed when mixing the single components. Hydrogels were formed from blends with an initial pH of 9 then upon addition of glucono-δ-lactone (GdL) forms gels at pH 3.3, as we have previously demonstrated that this procedure gives stiffer hydrogels.^23^

### Optimisation of 3D printing conditions

As previously discussed, to be suitable for extrusion-based 3D printing, a gel should exhibit thixotropy.^14^ At high shear rates, the gel should be able to easily flow through the syringe nozzle. As the material is printed onto a surface or substrate, its viscoelastic effects become important, and the kinetic energy applied during extrusion is converted into elastic energy and/or dissipated.^34^ Therefore, we first measured the recovery properties of PBI-A and PBI-A/PEO hydrogels using rheology (Fig. S1–S4, ESI†). A high shear rate (300%) was applied for 60 seconds to disrupt the equilibrated state of the gel, and hence break the network. The recoveries of *G*′ and *G*′′ were subsequently measured at a low shear rate (0.5%) for 200 seconds. The initial *G*′ and the percentage recovery of *G*′ are shown in Fig. S5, ESI.† Hydrogels formed from PBI-A alone showed a low recovery (7%). In comparison, hydrogels prepared from the PBI-A/PEO hydrogels had recoveries of 43%, 68%, and 45% (for 25/75, 50/50, and 75/25 blend ratios, respectively). Such results suggest that the polymer additive improves the thixotropic properties of the multicomponent hydrogel.

**Fig. 2:**
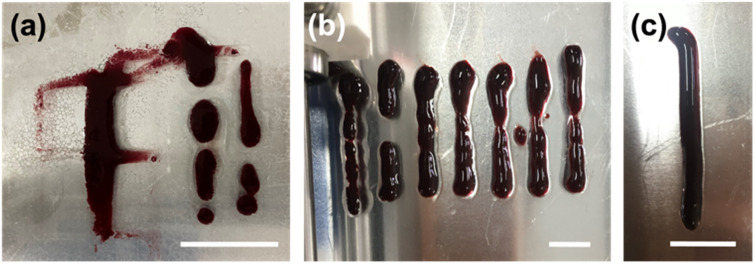
Photographs of (a) control PBI-A hydrogels printed at a total volume of 1000 μL, a nozzle height above the print bed of 3 mm, a nozzle speed relative to the print bed of 9408 mm min^−1^, and a printed line length of 6 cm. (b) Printed Gel-1 printed at a total volume of 1000 μL, a nozzle height above the print bed of 3 mm, and (from left to right) nozzle speeds relative to the print bed of 4704, 7056, 9408, 14 112, 18 881, 28 224 and 30 000 mm min^−1^. (c) Final optimised printed gel. Scale bar represents 2 cm.

After determining the recoverability using a shear recovery test on a rheometer, the hydrogel blends were all extrusion printed in 6 cm lines to correlate the recovery data to their printability (Fig. S6, ESI†). The shear rate at which a gel is extruded through the nozzle of the syringe can be calculated for a Newtonian fluid using eqn (1), where *γ* is the shear rate (in s^−1^), *V* is the volume of the extruded gel (in m^3^), *r* is the radius of the nozzle (in m), and *t* is the time taken to extrude the volume of the gel (in s). It is important to identify the shear rate applied during extrusion to implement the same conditions when measuring the rheological properties of the gels.^35^ Furthermore, the shear stress at any point within a sheared gel is determined by the value of the shear rate.^16^ Therefore, such information is critical to judge the material printability, printing resolution, and ink integrity.^34^ The automation of the 3D printer allows for precise control over the flow rate of the gel during extrusion, and eqn (1) was used to calculate the shear rate for all prints.1
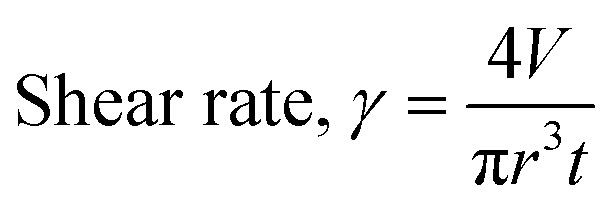
The 3D printer used has various parameters which can be optimised (such as the volume of the gel extruded, the extrusion speed, the height of the nozzle from the printing bed, and the translational speed of the extruder relative to the print bed).^2,36^ Each parameter was systematically changed and optimised by printing a line of 6 cm. The optimal value of the given parameter was that which gave the thinnest continuous line (examples given in Fig. 2b and Fig. S7 and S8, ESI†). A hydrogel of PBI-A only was also printed (Fig. 2a), with the gel completely breaking upon extrusion. These results suggest that the polymer is a prerequisite for creating thixotropic hydrogels suitable for printing continuous lines. The final optimal printing parameters are shown in Table S3, ESI†.

The gels formed from the PBI-A/PEO 50/50 blend had the highest recovery (68%, Fig. S5, ESI†), but when they were printed, they showed gaps in the lines (Fig. S6b, ESI†). Such results suggest that recovery tests alone are not enough to determine the printability of a hydrogel despite this being a commonly used proof of concept in the literature. From this preliminary screening, we chose to print hydrogels from the PBI-A/PEO 25/75 blend, as these gels gave the thinnest continuous lines when printed (Fig. S6a, ESI†). Here, this blend will be referred to as Gel-1 or Printed Gel-1 for simplicity.

### Using rheology to understand the printing process

As previously mentioned, rheology can be used to study the effects of applied shear on the restoration kinetics of the gel. Although recovery tests can inform one about the printability of hydrogels, they may not be representative of the extrusion process despite being used as a proxy for this. Therefore, we carried out a series of experiments using rheology to try and replicate the gelling conditions and printing conditions that would be experienced by the gel in a syringe.

**Fig. 3:**
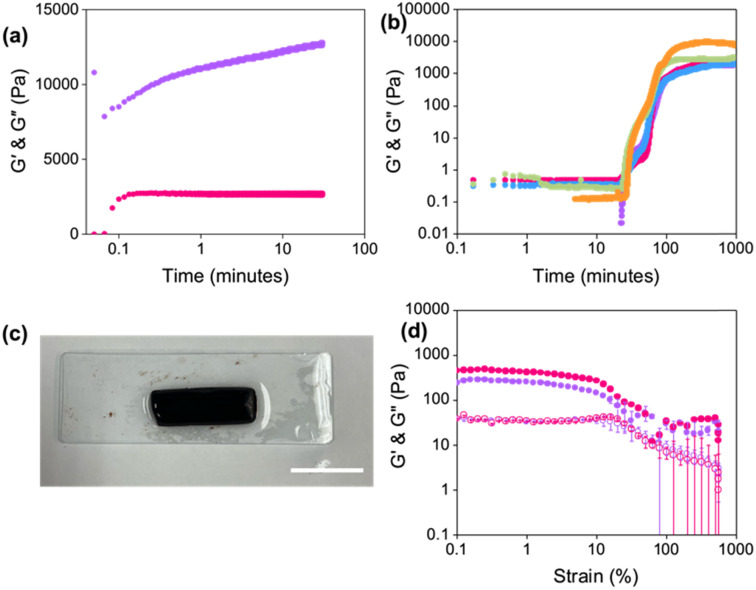
(a) Restoration of *G*′ (purple) and *G*′′ (pink) as a function of time after shearing Gel-1 at 2500 s^−1^ for 1 second. (b) Development of *G*′ during the gelation of Gel-1 under a normal force of 0 N (purple), 0.10 N (pink), 0.15 N (blue), 0.20 N (green), and 0.50 N (orange). Measurements were performed under a strain of 0.5%, a frequency of 10 rad s^−1^, and at 25 °C. (c) Photograph of a slice of Gel-1 gelled inside a syringe. Scale bar represents 2 cm. (d) Strain sweeps of syringe gels formed at the top (purple) and bottom (pink) of the syringe. Closed circles represent *G*′ and open circles represent *G*′′. Data shown are averaged data for triplicate runs, with error bars representing standard deviation.

To try and mimic the shear applied during printing, the hydrogel was exposed to 2500 s^−1^ (as calculated using eqn (1)) for 1 second. *G*′ and *G*′′ were then monitored over time (Fig. 3a). Both *G*′ and *G*′′ initially dropped in value, which could be due to slight slippage during the shearing process. After 1 minute, the moduli were the same value as those for the pre-sheared gel. The gels continued to show a stepwise increase in *G*′ over time. However, the gels were only slightly different to the original pre-sheared sample (*G*′ values of 10 800 and 12 800 Pa for the pre-sheared and sheared samples, respectively) and suggests that the gels are not broken upon extrusion. However, it should be emphasised that such measurements are not fully representative of the type of shear found inside a syringe during extrusion.

Since the gels were formed in 3.5 mL syringes and left to gel overnight, we hypothesised that pre-compression of the gel could potentially occur inside the syringe, leading to strengthening of the network.^2^ It has previously been reported that compression of gels resulted in non-reversible changes to the networks of similar LWMGs.^37^ Therefore, a compression sweep was carried out where the gap distance of the measuring system was lowered from a position of 1.8 mm by 5 μm s^−1^ for 5 minutes. After this time, the measuring system was lifted back to 1.8 mm, and a strain sweep was immediately run (Fig. S9, ESI†). Compressed gels were stiffer (*G*′ values at 0.01% strain of 1380 and 31 200 Pa before and after compression, respectively), in agreement with previous data on similar pH-triggered gels.^2,37^ However, the compressed gels were significantly weaker than the non-compressed gels, differing from previous reports. The yield points of the gels were 3.2% and 0.02% before and after compression, respectively. This change in strength suggests that the fibres may interact and entangle differently or that different types of fibres are formed upon compression. However, another possible explanation could be that as the strain sweeps were performed immediately after compression, the gels did not have time to recover fully.

Next, kinetic measurements of the gelation process were performed under different normal forces, *F*, measuring the development in *G*′ and *G*′′ over time (Fig. 3b) to mimic the force experienced in the syringe. The *G*′ values of the hydrogels at 1000 minutes increased with increasing normal force applied, indicating that the gels were becoming stiffer. However, the development of the moduli was similar irrespective of the force, suggesting that the gels all undergo the same self-assembly process. Such results suggest that compression of the gels strengthens the network, which may result in a gradient effect in the printing results if one is printing large volumes of gel from a single syringe.

To determine whether there was a gradient effect on the gels formed at different depths of the syringe, strain sweeps were conducted on slices of gels from the same syringe (Fig. 3c). Gels were again formed in 3.5 mL syringes with the nozzle removed. The gels were then sliced in half to compare the rheological properties of the gel formed at the top and bottom of the syringe. The value of *G*′ nearly doubled when comparing the gel from the bottom of the syringe to that from the top (Fig. 3d, *G*′ values at 0.1% strain of 440 Pa and 240 Pa for the bottom and top of the syringe, respectively). Furthermore, both gels had the same yield point of 1.6%. The linear viscoelastic region of both gels showed slight fluctuations in *G*′ values, which could be the result of loading artefacts. Comparing these results to Fig. S13, ESI,† would suggest that the force applied by the gel’s own weight in the syringe is minimal, giving more homogeneous gels. However, such results may not be observed when using larger syringes, which require greater volumes of gel.

### Characterisation of 3D printed hydrogels

**Fig. 4:**
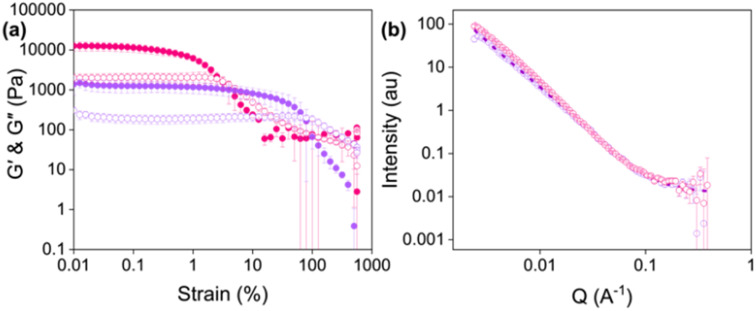
(a) Strain sweeps of Gel-1 (purple) and Printed Gel-1 (pink). Closed circles represent *G*′ and open circles represent *G*′′. Data shown are averaged data for triplicate runs, with error bars representing standard deviation. (b) Small-angle neutron scattering patterns for Gel-1 (purple) and Printed Gel-1 (pink). Open circles show the data and dashed lines represent the fit.

To characterise the properties of printed hydrogels, gels were printed directly into Sterilin vials. Printed gels were stiffer than the corresponding non-printed gels (Fig. 4a, *G*′ values at 0.01% strain of 12 550 Pa and 1380 Pa for printed and non-printed gels, respectively), which is at odds with results previously reported for similar LMWGs.^13,17^ Furthermore, the non-extruded gels were stronger, with a higher yield point (2.5% compared to 0.1% for the extruded gels). These changes in rheological properties could be due to the shear applied when the gels are extruded, or compression caused by the syringe plunger during extrusion altering the fibres or gel network. It is also possible that the energy dissipated in the system when sheared is causing an increase in gel stiffness.^38^

To elucidate what was happening to the fibre-level assembly upon printing, SANS was used to probe the gel structures before and after printing (Fig. 4b, Tables S4 and S5, ESI†). SANS is used to investigate the materials at the nanoscale.^39,40^ Neutrons are directed at a sample, and how these neutrons scatter at small angles due to interactions with atomic nuclei is measured. This scattering provides information about the size, shape, and arrangement of the structures present in the sample. Therefore, this technique was used to determine whether the gel fibres are altered by extrusion. The scattering of gels prepared directly into cuvettes (non-extruded) was compared to those prepared in syringes and extruded into the cuvettes before measurement.

The scattering data fit to an elliptical cylinder with a power law before and after extrusion. The scattering intensity at low *Q* (0.002 < *Q* < 0.01) increased upon extrusion, suggesting an increase in the number of large self-assembled structures and loss of homogeneity after printing. Furthermore, the axis ratio significantly increases for extruded hydrogels (5.2 *versus* 1.8 for printed and non-printed gels, respectively). An increase in axis ratio indicates that the fibres are more compact or tape-like in the extruded gels,^24,41^ and could explain the increase in stiffness observed in the rheological data (Fig. 4a). One explanation could be that the extrusion process pushes the fibres into a more continuous network, and the compression causes them to elongate.

For the printed gels to be suitable for applications, the gels should be homogeneous along the printing axis. Therefore, cavitation rheology was utilised to measure the critical pressure at different points along the printed gel (Fig. S11, ESI†). This technique is a form of microrheology which utilises the cavitation effect to probe the localised mechanical properties of a material.^42^ Cavitation rheology has the advantage that it can be conducted on gels of any shape in their native environment.^43,44^ The critical pressure was measured in 0.5 cm increments along the length of the printed gel, with a control experiment performed in a Sterilin vial (Fig. S15, ESI†). The critical pressure was identical at points 1.0 and 1.5 cm along the printing axis (22 Pa for 1.0 and 1.5 cm, respectively) and was slightly lower (16 Pa) 0.5 cm along the gel. This decrease in pressure could be due to this section of gel being from the top of the syringe, so it is not as compressed as the gel from the middle and bottom of the syringe, in agreement with the rheology data.

### Using RheoSANS to understand the printing process

To investigate the structural changes during different stages of the printing process, we employed *in situ* RheoSANS (simultaneous rheology and small-angle neutron scattering). We chose neutron scattering over X-ray scattering because SANS is more suited to studying *in situ* processes occurring over several hours due to better penetration of neutrons compared to X-rays. While RheoSANS has been extensively used to monitor various gelling systems,^45–48^ no studies, to the best of our knowledge, have been reported on characterising printed hydrogels. Combining rheology with *in situ* SANS allows us to correlate changes in stiffness during printing (probed by rheology) to changes in fibril structures (which can be probed by SANS).

**Fig. 5:**
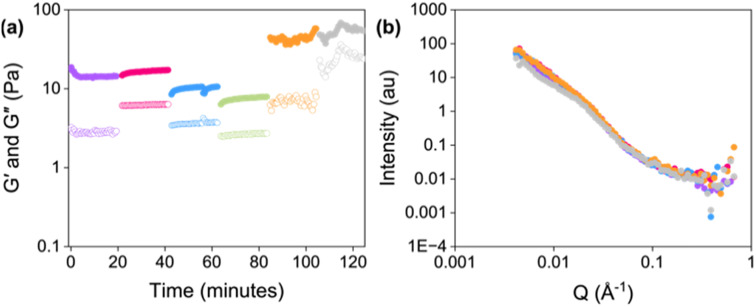
(a) Change in *G*′ and *G*′′ with increasing shear applied. Closed circles represent *G*′ and open circles represent *G*′′. (b) Small-angle neutron scattering patterns for Gel-1. Open circles show the data and dashed lines represent the fit. The gel was sheared at shear rates of 0 (purple), 1 (pink), 10 (blue), 100 (green), 1000 (orange), and 2500 (grey) rad s^−1^.

We first ran a kinetic measurement to collect rheology and scattering data as the gel formed (Fig. S12a and Tables S6–S10, ESI†). As this measurement uses a titanium concentric cylinder geometry to allow neutrons to pass through the sample, the absolute *G*′ and *G*′′ will be affected by the geometry used.^48^ However, the observed trends remained consistent when using a parallel plate geometry (Fig. S13, ESI†). After gelation, the gel was sheared from 1 to 2500 rad s^−1^, with each cycle’s shear rate increasing by a factor of 10 from 1 to 1000 rad s^−1^, and then linearly increasing the shear rate to 2500 rad s^−1^ (Fig. 5a). The sample was held at the chosen shear for 20 minutes before transitioning to the next. The unsheared sample fit to an elliptical cylinder with a power law with a cylinder radius of 64 Å (Fig. 5b, Table S11, ESI†). At the lowest shear rates (1 and 10 rad s^−1^), the data still shows excellent fit to an elliptical cylinder and power law, with negligible changes in the fit parameters (Fig. 5b, Tables S12 and S13, ESI†). Similarly, *G*′ and *G*′′ values for the sample sheared at 1 rad s^−1^ were nearly identical to those of the unsheared gel (Fig. 5a, *G*′ values of 14 and 17 Pa, respectively). When the gel was sheared at 10 rad s^−1^, the *G*′ value slightly decreased to 9 Pa. However, at shear rates of 100 rad s^−1^ and above, the data now fit to a combined sphere and power law model (Fig. 5b, Tables S14–S16, ESI†). At 100 rad s^−1^, the *G*′ value decreased to 8 Pa. We noted at higher shear rates (1000 and 2500 rad s^−1^), there was a significant increase in *G*′ (45 and 65 Pa for 1000 rad s^−1^ and 2500 rad s^−1^, respectively), in agreement with the rheology data for the printed gels. These findings suggest that higher shear rates modify the structure of the fibres, leading to an increase in stiffness.

To mimic printing gels over longer time periods (*e.g.*, when printing patterns), we also ran *in situ* RheoSANS experiments where the gels were sheared at the same shear rate for three cycles (Fig. S14 and Table S17, ESI†). We used a shear rate of 2500 rad s^−1^, calculated using eqn (1). After the first shear cycle, the data again fit to a combined sphere and power law model. However, after each shear cycle, there were minimal changes in the scattering data, suggesting that extending the duration of printing is unlikely to significantly affect the resulting gel structures.

**Fig. 6:**
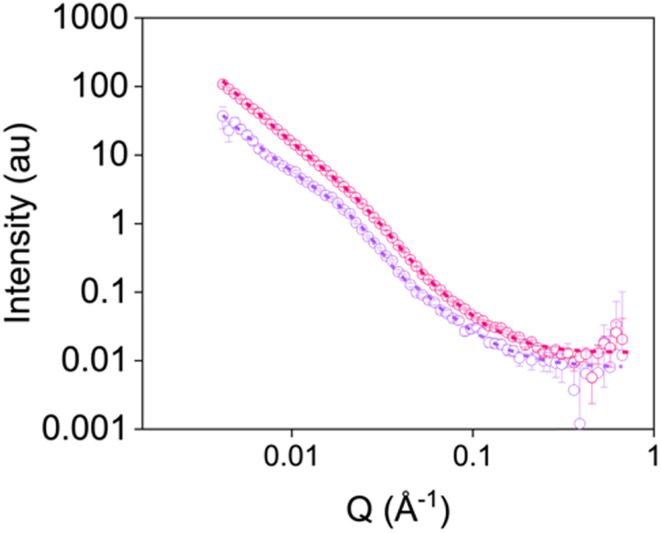
Small-angle neutron scattering patterns for gels sheared at 2500 s^−1^ using the rheometer (purple) and a syringe pump (pink). Open circles show the data and dashed lines represent the fit.

To determine whether rheological data can be correlated to printed gels, we also ran static SANS measurements on gels which had been sheared using a syringe pump (examples given in Fig. 6 and Fig. S16–S19 and Tables S19–S23, ESI†). Again, gels were formed in syringes. Once gelled, the samples were extruded into cuvettes at the different shear rates applied on the rheometer. Comparing data from the RheoSANS experiment to these static measurements, there were differences in the fibre structures formed after application of the higher shear rates (Fig. 5b and Fig. S18–S19, Tables S14–S16 and S21–S23, ESI†). All data for gels sheared at rates above 100 rad s^−1^ on the rheometer fit to the combined sphere and power law model (Fig. 6 and Tables S14–S16, ESI†); this suggests that the fibres have been significantly disrupted and have not reformed. In comparison, when a syringe pump was used at these shear rates, the data still fit to an elliptical cylinder and power law model (Fig. 6 and Fig. S18–S19 and Tables S21–S23, ESI†) suggesting these fibres remain intact. Such results suggest that one cannot necessarily correlate data for gels sheared by the rheometer to those sheared using different methods, emphasising that care must be taken when characterising these systems.

## Conclusions

In conclusion, we have developed a comprehensive series of rheological and scattering techniques to characterise LMWG-based hydrogels before, during, and after 3D printing. This work highlights the importance of thorough characterisation at each stage of the printing process, across multiple length scales, to fully understand how printing affects these materials. We demonstrate that rheological recovery tests, the most used technique to suggest material printability, are not sufficient when put into practice. Through comparing the structures formed after shearing and printing with rheology and SANS we have shown the difference in mechanical properties and morphological properties of the fibres and the network. Through this we highlight that care must be taken when characterising these systems, as gels sheared by different methods will have different properties. Therefore, we hope the work presented here may act as an aid in the characterisation of new materials made using 3D printing and would suggest, as always, that characterisation should carried out on materials as intended for end use and as *in situ* as possible.

## Author contributions

Conceptualisation: RG, ED; methodology: RG, JD, ED; validation: RG; formal analysis: RG; investigation: RG, JD, ED; data curation: RG; visualisation: RG; supervision: ED; project administration: ED; funding acquisition: ED. All authors contributed to the writing of the manuscript.

## Conflicts of interest

There are no conflicts to declare.

## Supplementary Material

FD-260-D4FD00185K-s001

## Data Availability

Processed data to support this work is provided in the ESI.† Raw data available from the authors upon request. Unfitted and corrected SANS data is available *via* the ISIS Neutron and Muon Source Data Journal as DOI: https://doi.org/10.5286/ISIS.E.RB2310032-1 and https://doi.org/10.5286/ISIS.E.RB2410208-1.

## References

[cit1] Prendergast M. E., Burdick J. A. (2020). Adv. Mater..

[cit2] Nolan M. C., Caparrós A. M. F., Dietrich B., Barrow M., Cross E. R., Bleuel M., King S. M., Adams D. J. (2017). Soft Matter.

[cit3] Liu C., Xu N., Zong Q., Yu J., Zhang P. (2021). Colloid Interface Sci. Commun..

[cit4] Omar J., Ponsford D., Dreiss C. A., Lee T., Loh X. J. (2022). Chem.–Asian J..

[cit5] Chalard A., Mauduit M., Souleille S., Joseph P., Malaquin L., Fitremann J. (2020). Addit. Manuf..

[cit6] Puza F., Lienkamp K. (2022). Adv. Funct. Mater..

[cit7] Li J., Xing R., Bai S., Yan X. (2019). Soft Matter.

[cit8] Shi L., Carstensen H., Hölzl K., Lunzer M., Li H., Hilborn J., Ovsianikov A., Ossipov D. A. (2017). Chem. Mater..

[cit9] Suntornnond R., An J., Chua C. K. (2017). Macromol. Mater. Eng..

[cit10] Ouyang L., Highley C. B., Rodell C. B., Sun W., Burdick J. A. (2016). ACS Biomater. Sci. Eng..

[cit11] Sapuła P., Bialik-Wąs K., Malarz K. (2023). Pharmaceutics.

[cit12] Susapto H. H., Alhattab D., Abdelrahman S., Khan Z., Alshehri S., Kahin K., Ge R., Moretti M., Emwas A.-H., Hauser C. A. E. (2021). Nano Lett..

[cit13] Hill M. J. S., Adams D. J. (2022). Soft Matter.

[cit14] Champeau M., Heinze D. A., Viana T. N., de Souza E. R., Chinellato A. C., Titotto S. (2020). Adv. Funct. Mater..

[cit15] Schwab A., Levato R., D’Este M., Piluso S., Eglin D., Malda J. (2020). Chem. Rev..

[cit16] Herrada-Manchón H., Fernández M. A., Aguilar E. (2023). Gels.

[cit17] Fuentes-Caparras A. M., Canales-Galarza Z., Barrow M., Dietrich B., Laüger J., Nemeth M., Draper E. R., Adams D. J. (2021). Biomacromolecules.

[cit18] Panja S., Adams D. J. (2019). Chem. Commun..

[cit19] Panja S., Dietrich B., Adams D. J. (2020). ChemSystemsChem.

[cit20] Randle R. I., Ginesi R. E., Matsarskaia O., Schweins R., Draper E. R. (2023). Macromol. Rapid Commun..

[cit21] Duraisamy D. K., Reddy S. M. M., Saveri P., Deshpande A. P., Shanmugam G. (2024). Macromol. Rapid Commun..

[cit22] Thomson L., Ginesi R. E., Osborne D. D., Draper E. R., Adams D. J. (2023). Chem.–Eur. J..

[cit23] Ginesi R. E., Murray N. R., Dalgliesh R. M., Doutch J., Draper E. R. (2023). Chem.–Eur. J..

[cit24] Welsh T. A., Egan J. G., Dietrich B., Rafferty N., Ginesi R. E., Doutch J., Schweins R., Draper E. R. (2024). JPhys Mater..

[cit25] Draper E. R., Archibald L. J., Nolan M. C., Schweins R., Zwijnenburg M. A., Sproules S., Adams D. J. (2018). Chem.–Eur. J..

[cit26] Draper E. R., Walsh J. J., McDonald T. O., Zwijnenburg M. A., Cameron P. J., Cowan A. J., Adams D. J. (2014). J. Mater. Chem. C.

[cit27] McDowall D., Greeves B. J., Clowes R., McAulay K., Fuentes-Caparrós A. M., Thomson L., Khunti N., Cowieson N., Nolan M. C., Wallace M., Cooper A. I., Draper E. R., Cowan A. J., Adams D. J. (2020). Adv. Energy Mater..

[cit28] Cameron J., Adams D. J., Skabara P. J., Draper E. R. (2022). J. Mater. Chem. C.

[cit29] Andriamiseza F., Bordignon D., Payré B., Vaysse L., Fitremann J. (2022). J. Colloid Interface Sci..

[cit30] Draper E. R., Greeves B. J., Barrow M., Schweins R., Zwijnenburg M. A., Adams D. J. (2017). Chem.

[cit31] Pont G., Chen L., Spiller D. G., Adams D. J. (2012). Soft Matter.

[cit32] Hanabusa K., Itoh A., Kimura M., Shirai H. (1999). Chem. Lett..

[cit33] Adhia Y. J., Schloemer T. H., Perez M. T., McNeil A. J. (2012). Soft Matter.

[cit34] Bercea M. (2023). Molecules.

[cit35] Hernandez H. L., Souza J. W., Appel E. A. (2021). Macromol. Biosci..

[cit36] Kyle S., Jessop Z. M., Al-Sabah A., Whitaker I. S. (2017). Adv. Healthcare Mater..

[cit37] Adams D. J., Mullen L. M., Berta M., Chen L., Frith W. J. (2010). Soft Matter.

[cit38] Li Y., Zhu C., Dong Y., Liu D. (2020). Polymer.

[cit39] Gommes C. J., Jaksch S., Frielinghaus H. (2021). J. Appl. Crystallogr..

[cit40] McDowall D., Adams D. J., Seddon A. M. (2022). Soft Matter.

[cit41] Patterson C., Dietrich B., Wilson C., Mount A. R., Adams D. J. (2022). Soft Matter.

[cit42] Hill M. J. S., Fuentes-Caparrós A. M., Adams D. J. (2023). Biomacromolecules.

[cit43] Fuentes-Caparrós A. M., Dietrich B., Thomson L., Chauveau C., Adams D. J. (2019). Soft Matter.

[cit44] Zimberlin J. A., Sanabria-DeLong N., Tew G. N., Crosby A. J. (2007). Soft Matter.

[cit45] Eberle A. P. R., Porcar L. (2012). Curr. Opin. Colloid Interface Sci..

[cit46] Blair D. L. , in Molecular Gels: Structure and Dynamics, Royal Society of Chemistry, Cambridge, 2018, pp. 28–56

[cit47] Riley J. K., Richards J. J., Wagner N. J., Butler P. D. (2018). Soft Matter.

[cit48] McAulay K., Thomson L., Porcar L., Schweins R., Mahmoudi N., Adams D. J., Draper E. R. (2020). Org. Mater..

