# Supplementary material for: Can we mimic 3D printing of low molecular weight gels using a rheometer? – a characterisation toolkit for extrusion printed gels
Source: Faraday Discuss. 2024 Dec 9;260:347–59. doi: 10.1039/d4fd00185k (PMC12076186; doi:10.1039/d4fd00185k)
Supplement: FD-260-D4FD00185K-s001 [file FD-260-D4FD00185K-s001.pdf]

Supplementary Information (SI) for Faraday Discussions.
This journal is © The Royal Society of Chemistry 2025

Supporting Information

1. Experimental Methods

Materials. PBI-A was synthesised as reported previously.[1] All commercial reagents were used as received. Perylene-3,4-9,10-tetracarboxylic dianhydride (PTCDA), L-alanine, imidazole, and poly (ethylene oxide) (PEO, average M_n = 500,000) were purchased from Sigma-Aldrich. NaOD was purchased from Sigma-Aldrich as a 40 wt% solution in D_2O and diluted with D_2O to provide a 0.1 M solution.

Preparation of PBI-A/PEO blends. Stock solutions of **PBI-A** and **PEO** were made separately in deionised water at concentrations of 6.66 mg/mL, 10 mg/mL, and 20 mg/mL, and combined in their appropriate ratios to give blends with final concentrations of 5 mg/mL of each component. For **PBI-A** solutions, an equimolar equivalent of sodium hydroxide (0.1 M, aqueous) was added. The solutions were agitated on a MX-T6-S tube roller (SCILOGEX) overnight until all the gelator had visibly dissolved. The **PBI-A** solution was then adjusted to pH 9 using NaOH (1 M, aqueous). For 25:75 (v/v) blends, 2.5 mL of **PBI-A** (20 mg/mL) and 7.5 mL of **PEO** (6.66 mg/mL) were combined. For 50:50 (v/v) blends, 5 mL of **PBI-A** (10 mg/mL) and 5 mL of **PEO** (10 mg/mL) were added. For 75:25 (v/v) blends, 7.5 mL of **PBI-A** (6.66 mg/mL) and 2.5 mL of **PEO** (20 mg/mL) were added. The blends were left to mix overnight.

pH measurements. An FC200 pH probe from HANNA instruments with a 6 mm x 10 mm conical tip was used for pH measurements at 25°C. The state accuracy of the pH measurements is ± 0.1.

Preparation of PBI-A/PEO hydrogels. A pH-switch method was used to form the hydrogels. Solutions and blends were prepared as above. For non-printed hydrogels, 2 mL of solution was transferred to a 7 mL Sterilin vial containing a pre-weighed amount of glucono-δ-lactone (GdL) and shaken three times. The sample was left to stand overnight to allow gelation to occur. For printed hydrogels, 2 mL of solution was transferred to a 7 mL Sterilin vial containing a pre-weighed amount of GdL and gently shaken three times. The mixture was quickly transferred to a 3.5 mL syringe, and the syringe nozzle was tightly wrapped in parafilm to prevent the gel from drying out. Syringes were stood vertically on their plungers and left overnight to gel.

3D printing. Gels were prepared in 3.5 mL syringes as described above. A 3D printer (RepRap Ormerod 1) was modified and repurposed for gel printing by Dr Bart Dietrich (University of Glasgow).[2] The inner diameter of the 3.5 mL syringe nozzles used for extrusion was 2.2 mm. To print a 6 cm gel line at a shear rate of 2500 s^{-1} and a rate of extrusion of 166 μL/cm, the speed of the printer head was 9408 mm/min, raised 3 mm above the printing bed.

Rheology. Dynamic rheological measurements were performed with an Anton Paar Physica MCR301 rheometer. A cup-and-vane measuring system was used for recovery tests and strain sweeps, and a parallel plate measuring system for compression sweeps and time sweeps. Gels were prepared as described above. Printed gels were printed directly into Sterilin vials. The temperature was maintained at 25°C during all measurements using a water bath. All measurements were recorded in triplicate.

Recovery tests: For recovery tests, a constant frequency of 10 rad/s and strain of 0.5% were first applied for 200 seconds, followed by a strain of 300% for 60 seconds to destroy the gel. Restoration of G' and G" were monitored in the subsequent time sweep (with a frequency of 10 rad/s and a strain of 0.5%) for 200 seconds. The shear-recovery cycles were performed 5 times. The percentage recovery was calculated by dividing the average G' after each restoration by the original G'.

Recovery after extrusion: A parallel plate geometry (diameter of plate = 12.5 mm) was used for the constant shear and recovery measurements. As the parallel plate is not compatible with the cup measuring system, a 3D printed holder was used to secure the Sterilin vial in place. To mimic printing through a syringe, a constant shear of 2500 s^{-1} was applied to the sample for 1 second. After this time, G' and G" were measured at an angular frequency of 10 rad/s and with a strain of 0.5% for 30 minutes.

Strain sweeps: Strain sweeps were performed over a range of 0.1% to 1000% with a frequency of 10 rad/s. The critical strain (yield point) were quoted where G' departed from linearity and ultimately crossed over G" (flow point), causing the gel to break down.

Frequency sweeps: Frequency sweeps were performed from 1 rad/s to 100 rad/s under a strain of 0.1%. G' and G" were quoted at 10 rad/s. The measurements were performed within the viscoelastic region where G' and G" were independent of strain amplitude.

Compression sweeps: Compression sweeps were performed at a constant strain of 0.5% and a constant frequency of 10 rad/s. The gap distance was decreased from a position of 1.8 mm at a constant rate of 5 μm/s for 5 minutes. After this time, the parallel plate was raised back to a gap distance of 1.8 mm and a strain or frequency sweep measurement was performed.

Time sweeps under compression: Time sweeps were performed with a 50 mm sandblasted plate and a plate gap of 0.8 mm. Tests were performed at an angular frequency of 10 rad/s and with a strain of 0.5%. The normal force was set at either 0, 0.1, 0.15, 0.2, or 0.5 N. Mineral oil was carefully added around the circumference of the top plate to prevent the sample from drying out.

SANS. SANS measurements were performed using the SANS2D instrument (ISIS, Rutherford Appleton Laboratories, Didcot, UK) under experiment numbers RB2310032 and RB2410208. Measurements were performed using a wavelength band of 0.9 to 13 Å to access a Q range of 0.004 to 0.7 $Å^{-1}$. Solutions and gels were prepared in 2 mm path length UV spectrophotometer quartz cuvettes (Hellma). These were placed in a temperature-controlled sample rack during the

measurements. Measurements were ran at 25°C. Solutions were prepared as described above, but in D_2O and NaOD (0.1 M). For non-printed gels, the pre-gel solutions were added to Sterilin vials with the appropriate amount of GdL, shaken three times, and quickly transferred to the cuvettes before leaving to gel overnight. For printed gels, gels were prepared in 3.5 mL syringes and left to gel overnight, with syringes stood vertically on their plungers. These gels were then transferred into the cuvette using a syringe pump (Aladdin-220 Syringe Pump, World Precision Instruments) set at different flow rates.

The data were then reduced to 1D scattering curves of intensity vs. Q using the facility-provided software. The electronic background was subtracted, the full detector images for all data were then normalised, and the scattering from the empty cell was subtracted. The scattering from D_2O was also measured and subtracted from the data using the Mantid software package installed inside the ISIS virtual machines, IDAaaS.[3] The instrument-independent data were then fitted to the models discussed in the text using the SasView software package (version 5.0.4).[4] The scattering length density (SLD) of each material was calculated using the National Institute of Standards and Technology's neutron activation and scattering calculator.[5] The SLD of D_2O was calculated to be 6.393×10^{-6} Å^{-2}, the SLD of **PBI-A** was calculated to be 3.445×10^{-6} Å^{-2}, and the SLD of **PEO** was calculated to be 1.122×10^{-6} Å^{-2}. The SLD of the **PBI-A/PEO** blend was calculated to be 1.703×10^{-6} Å^{-2}. All data fit best to a cylindrical model combined with a power law. The best fit was determined as the one which overlapped well with the data and had the lowest χ^2 value.

RheoSANS. RheoSANS experiments were carried out using the SANS2D instrument (ISIS, Rutherford Appleton Laboratories, Didcot, UK) under experiment number RB2410208. Scattering data were collected with an 8 mm incident beam, a sample-to-detector distance of 4.0 m, and a wavelength range of 1.75-16.5 Å, resulting in a wave vector range of $0.007 \leq q \leq 1.5$ Å^{-1}. Rheology was collected on an Anton Paar Physica MCR 501 rheometer with a customised titanium concentric cylinder (ME49-1.16-60/108.5/T) and a temperature controller (TC-30) in the radial direction with a gap of 1 mm. Gelation was measured over time at a set strain of 0.5% and 10 rad/s at 25°C. A shear ramp experiment was performed under 4 shear rates (1, 10, 100, and 1000 rad/s), each applied for 1 second. The G' and G" values were measured under a strain of 0.5% and a frequency of 10 rad/s for 20 minutes after the application of each shear. For cycling experiments, the chosen shear rate was applied for 1 second and the G' and G" values were then measured under a strain of 0.5% and a frequency of 10 rad/s for 20 minutes. The shear-recovery cycles were performed 3 times. Scattering data was then reduced and fit as described above.

2. Supplementary Figures and Tables

Table S1. Rheology of gels formed from **PBI-A**/polymer blends.

Sample	G′ (Pa)	G" (Pa)	Yield Point (%)	Flow Point (%)	tanδ
**PBI-A/PEG 25/75**	570	92	2.00	63.1	0.16
**PBI-A/PEG 50/50**	1048	187	2.51	39.8	0.18
**PBI-A/PEG 75/25**	1370	244	2.51	251	0.18
**PBI-A/PEO 25/75**	876	124	31.6	794	0.14
**PBI-A/PEO 50/50**	801	148	10	100	0.19
**PBI-A/PEO 75/25**	1089	164	5.1	501	0.15
**PBI-A/PVA 25/75**	1553	228	2.51	501	0.15
**PBI-A/PVA 50/50**	2016	299	3.16	501	0.15
**PBI-A/PVA 75/25**	2923	466	2.00	501	0.16

Table S2. Concentration of glucono-δ-lactone required to form hydrogels from different **PBI-A/PEO** blends with a final pH of approximately 3.2. pH data shown are averaged data for triplicate samples, with errors representing standard deviation.

PBI-A/PEO blend	GdL concentration (mg/mL)	Average pH
25/75	12.5	3.29 ± 0.010
50/50	10	3.17 ± 0.010
75/25	12.5	3.24 ± 0.005

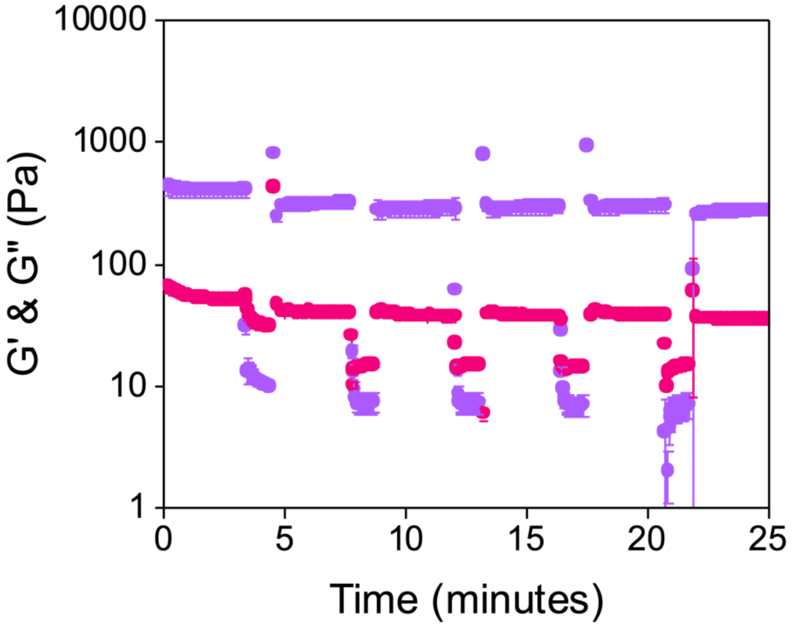

Figure S1. Rheological recovery test for gels made from a **PBI-A** solution. The gels were subjected to a constant frequency of 10 rad/s and a strain of 0.5% for 200 seconds, followed by a higher strain of 300% for 60 seconds. These cycles were repeated 5 times. Purple circles represent G′ and pink circles represent G″. Data shown are averaged data for triplicate runs of the samples, with error bars representing standard deviation.

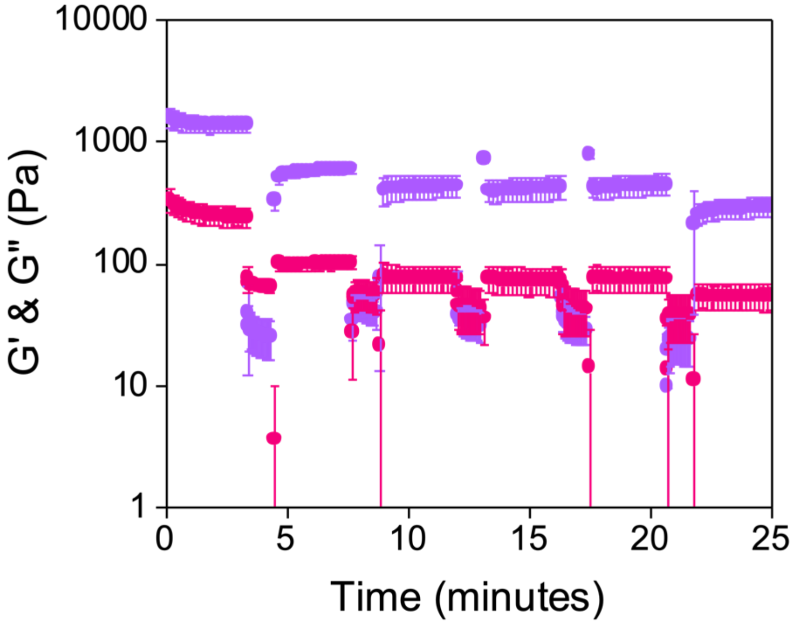

Figure S2. Rheological recovery test for gels made from a 25/75 **PBI-A/PEO**. The gels were subjected to a constant frequency of 10 rad/s and a strain of 0.5% for 200 seconds, followed by a higher strain of 300% for 60 seconds. These cycles were repeated 5 times. Purple circles represent G′ and pink circles represent G″. Data shown are averaged data for triplicate runs of the samples, with error bars representing standard deviation.

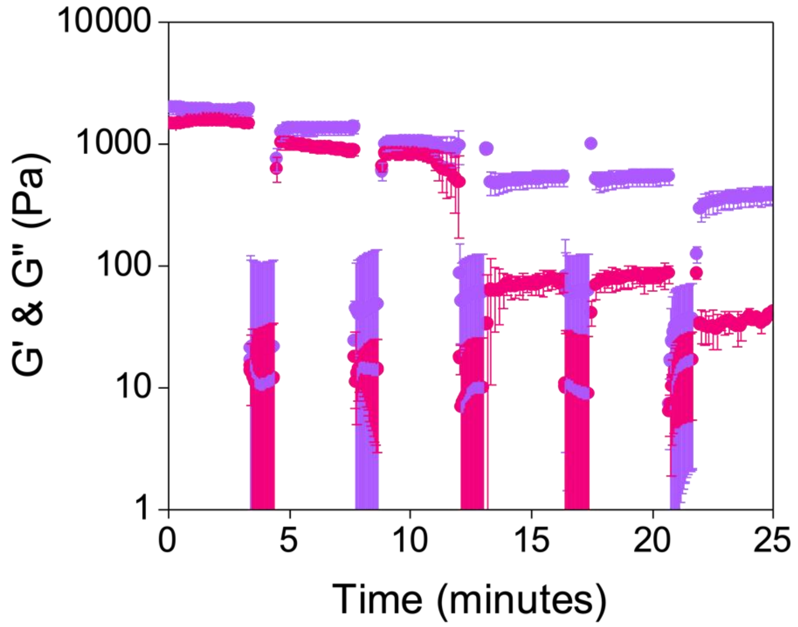

Figure S3. Rheological recovery test for gels made from a 50/50 **PBI-A/PEO** blend. The gels were subjected to a constant frequency of 10 rad/s and a strain of 0.5% for 200 seconds, followed by a higher strain of 300% for 60 seconds. These cycles were repeated 5 times. Purple circles represent G′ and pink circles represent G″. Data shown are averaged data for triplicate runs of the samples, with error bars representing standard deviation.

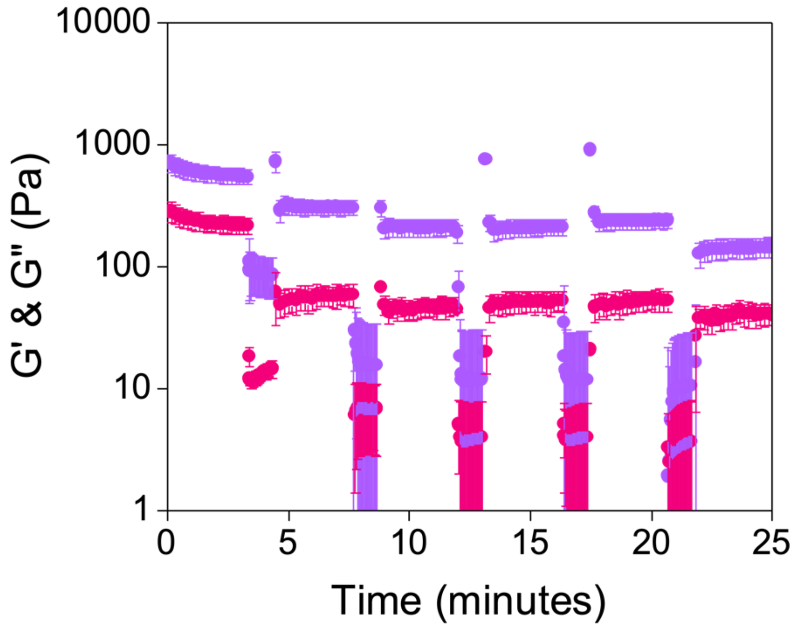

Figure S4. Rheological recovery test for gels made from a 75/25 **PBI-A/PEO** blend. The gels were subjected to a constant frequency of 10 rad/s and a strain of 0.5% for 200 seconds, followed by a higher strain of 300% for 60 seconds. These cycles were repeated 5 times. Purple circles represent G′ and pink circles represent G″. Data shown are averaged data for triplicate runs of the samples, with error bars representing standard deviation.

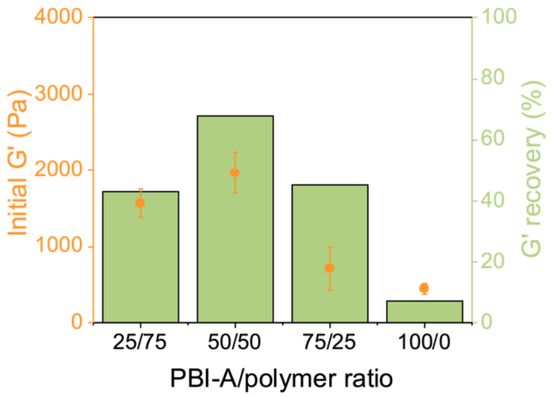

Figure S5. Rheology recovery tests of hydrogels from different **PBI-A**/PEO blends. The initial G′ (*i.e.*, the G′ value at t=0) is shown as orange circles. Data shown are averaged data for triplicate runs, with error bars representing standard deviation. The percentage recovery (green columns) is the ratios of the average G′ after each restoration with the original G′.

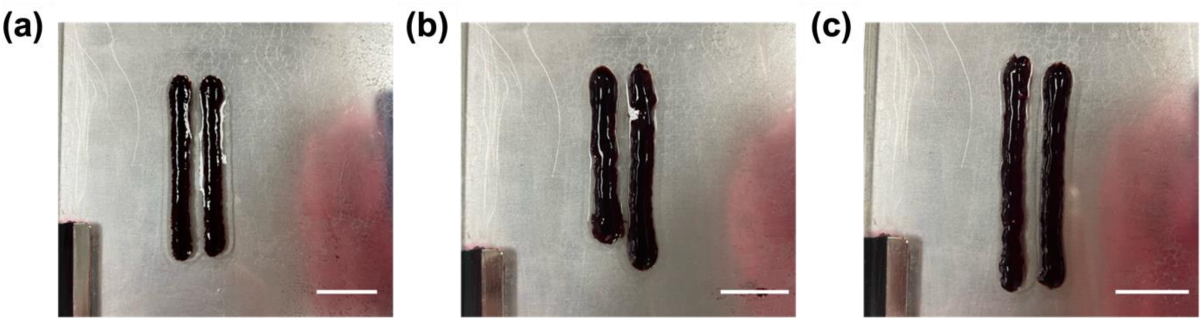

Figure S6. Photographs of printed gels from **PBI-A/PEO** (a) 25/75, (b) 50/50, and (c) 75/25 blends. Gels were printed at a total volume of 1000 µL, an accessory height of 3 cm, and a printing speed of 9408 mm/min. Scale bar represents 2 cm.

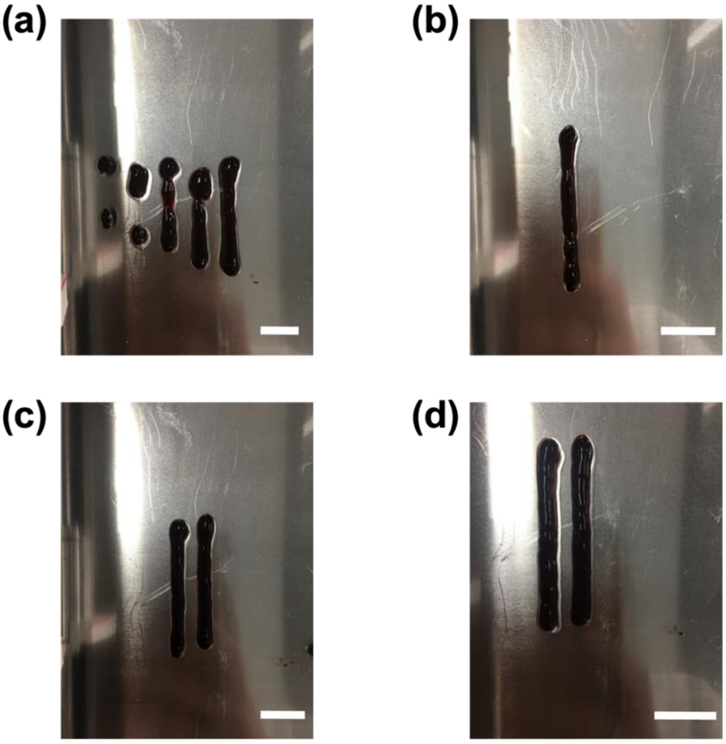

Figure S7. Photographs of **Printed Gel-1** hydrogels printed at a total volume of (a) from left to right, 100, 200, 300, 400, and 500 μL; (b) 600 μL; (c) from left to right, 700 and 800 μL; and (d) from left to right, 900 and 1000 μL. An accessory height of 3 cm and a speed of 9408 mm/min was used for all prints. Scale bar represents 2 cm.

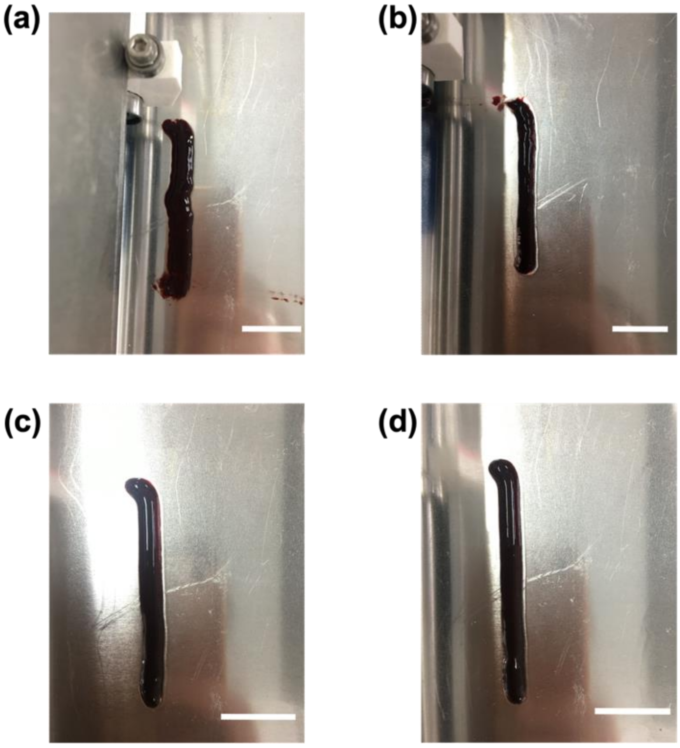

Figure S8. Photographs of **Printed Gel-1** hydrogels printed at an accessory height of (a) 1 cm, (b) 2 cm, (c) 3 cm, and (d) 4 cm from the printing bed. A total volume of 1000 μL and a speed of 9408 mm/min was used for all prints. Scale bar represents 2 cm.

Table S3. Optimal parameters for printing **Printed Gel-1** as shown in Figure 2c.

Printing parameter	Value
Total volume (μL)	1000
Volumetric rate (μL/cm)	166
Nozzle speed above bed (mm/min)	9408
Nozzle height from print bed (mm)	3
Time taken to print a 6 cm line (s)	0.38
Shear rate (s^{-1})	2500

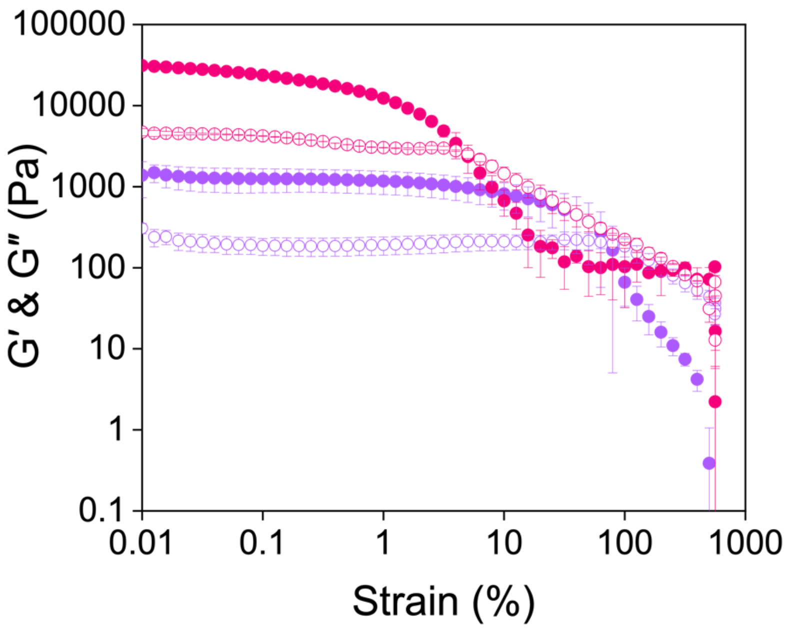

Figure S9. Strain sweep of **Gel-1** before (purple) and after (pink) compression. Closed circles represent G′ and open circles represent G″. Data shown are averaged data for triplicate runs, with error bars representing standard deviation.

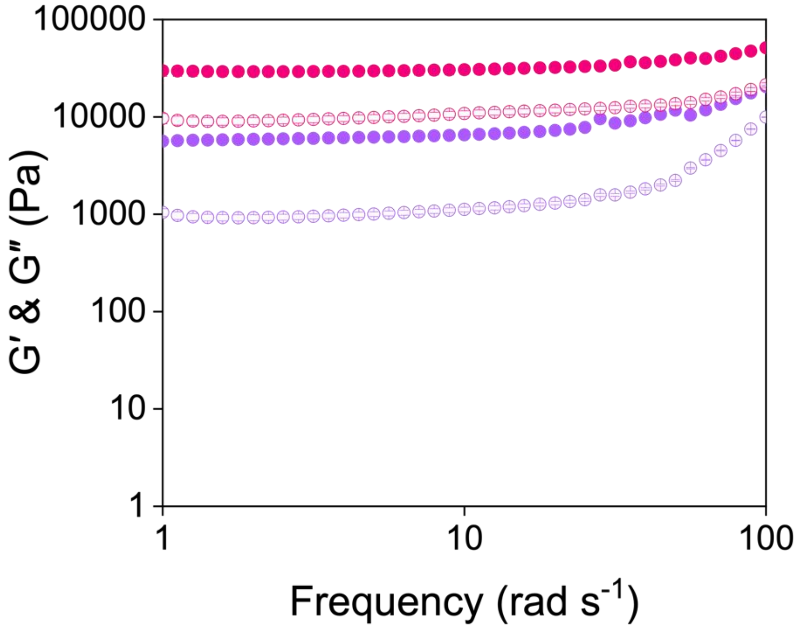

Figure S10. Frequency sweep of **Gel-1** before (purple) and after (pink) compression. Closed circles represent G′ and open circles represent G″. Frequency sweeps were performed at 0.1% strain at 25°C. Data shown are averaged data for triplicate runs, with error bars representing standard deviation.

Table S4. Tabulated parameters of the SANS model fit for **Gel-1**.

**Elliptical Cylinder + Power Law**	**Value**	**Error**
Background (cm^{-1})	0.01	
Cylinder scale	5.5860×10^{-5}	4.7674×10^{-6}
Length (Å)	600	215
Radius (Å)	50.0	1.8
Axis ratio	1.8	0.1
Power law scale	1.2938×10^{-4}	7.0703×10^{-6}
Power law	2.2	0.01
χ^2	1.6043	

Table S5. Tabulated parameters of the SANS model fit for **Printed Gel-1**.

Elliptical Cylinder + Power Law	Value	Error
Background (cm^{-1})	0.01	
Cylinder scale	1.1227×10^{-4}	7.4941×10^{-6}
Length (Å)	448	46
Radius (Å)	55.0	2.3
Axis ratio	5.2	0.4
Power law scale	7.1350×10^{-5}	3.7335×10^{-6}
Power law	2.3	0.01
χ^2	1.2917	

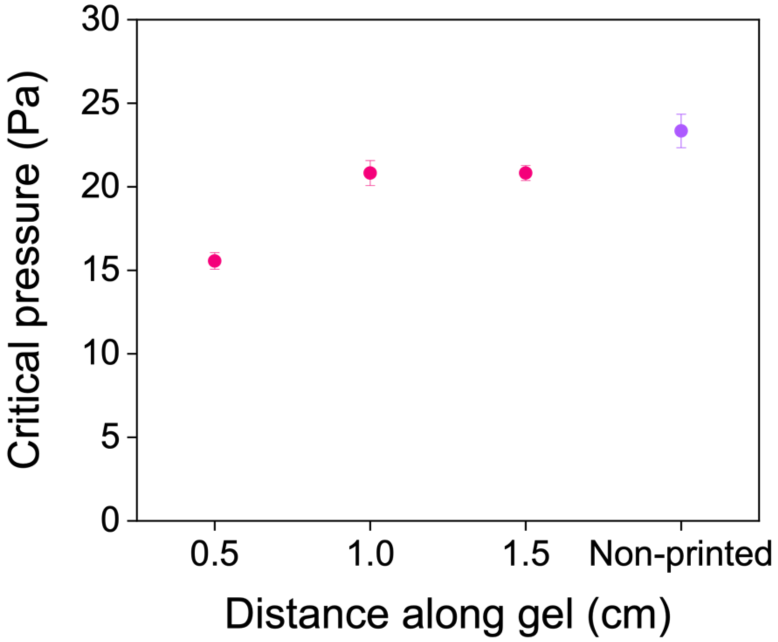

Figure S11. Cavitation rheology of **Gel-1** (purple) and **Printed Gel-1** (pink). Data shown are averaged data for triplicate runs, with error bars representing standard deviation.

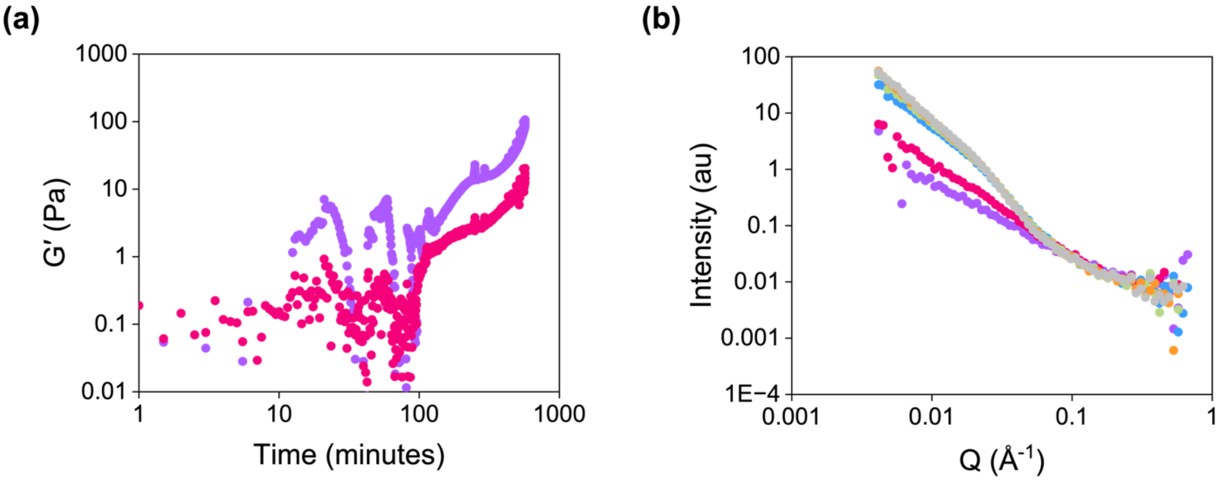

Figure S12. (a) Plot showing the evolution of the gel network of **Gel-1** in the RheoSANS experiment. The graph shows the development of G′ (purple) and G″ (pink) with time. (b) Small-angle neutron scattering patterns for **Gel-1** 0 (purple), 100 (pink), 200 (blue), 300 (green), 400 (orange), and 500 (grey) minutes after gelation was triggered.

Table S6. Tabulated parameters of the SANS model fit for **Gel-1** 100 minutes after gelation was triggered.

**Sphere + Power Law**	**Value**	**Error**
Background (cm^{-1})	0.004	
Sphere scale	2.6688×10^{-5}	3.9194×10^{-6}
Radius (Å)	76.7	4.0
Power law scale	5.6843×10^{-4}	4.6289×10^{-5}
Power law	1.6	0.02
χ^2	1.1143	

Table S7. Tabulated parameters of the SANS model fit for **Gel-1** 200 minutes after gelation was triggered.

**Elliptical Cylinder + Power Law**	**Value**	**Error**
Background (cm^{-1})	0.004	
Cylinder scale	2.7789×10^{-4}	1.0126×10^{-5}
Length (Å)	827	25
Radius (Å)	47.1	1.1
Axis ratio	2.3	0.1
Power law scale	7.7846×10^{-5}	5.2632×10^{-6}
Power law	2.3	0.02
χ^2	1.1767	

Table S8. Tabulated parameters of the SANS model fit for **Gel-1** 300 minutes after gelation was triggered.

**Elliptical Cylinder + Power Law**	**Value**	**Error**
Background (cm^{-1})	0.004	
Cylinder scale	2.0483×10^{-4}	8.2045×10^{-6}
Length (Å)	750	217
Radius (Å)	54.6	1.2
Axis ratio	1.9	0.1
Power law scale	5.7177×10^{-5}	3.3051×10^{-6}
Power law	2.4	0.01
χ^2	1.0074	

Table S9. Tabulated parameters of the SANS model fit for **Gel-1** 400 minutes after gelation was triggered.

**Elliptical Cylinder + Power Law**	**Value**	**Error**
Background (cm^{-1})	0.004	
Cylinder scale	2.2257×10^{-4}	7.5590×10^{-6}
Length (Å)	585	82
Radius (Å)	60.6	1.6
Axis ratio	1.8	0.1
Power law scale	4.5271×10^{-5}	2.1333×10^{-6}
Power law	2.5	0.01
χ^2	1.0458	

Table S10. Tabulated parameters of the SANS model fit for **Gel-1** 500 minutes after gelation was triggered.

**Elliptical Cylinder + Power Law**	**Value**	**Error**
Background (cm^{-1})	0.004	
Cylinder scale	2.0477×10^{-4}	6.8501×10^{-6}
Length (Å)	250	12
Radius (Å)	63.5	1.9
Axis ratio	1.8	0.1
Power law scale	3.2436×10^{-5}	1.6716×10^{-6}
Power law	2.6	0.01
χ^2	1.1157	

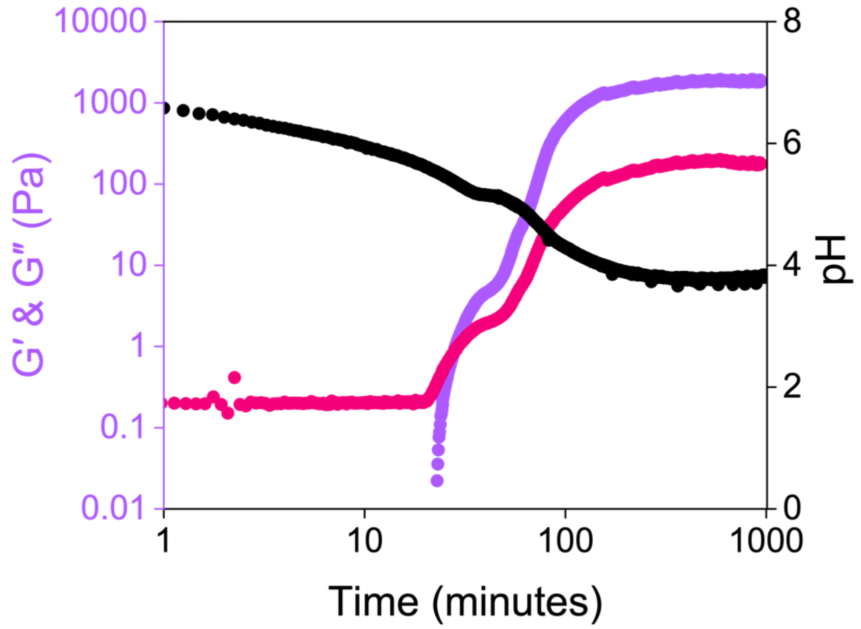

Figure S13. Plot showing the evolution of the gel network of **Gel-1**. The graph shows the development of G′ (purple) and G″ (pink) with time and change in pH (black).

Table S11. Tabulated parameters of the SANS model fit for an unsheared sample of **Gel-1.**

**Elliptical Cylinder + Power Law**	**Value**	**Error**
Background (cm^{-1})	0.004	
Cylinder scale	2.0477×10^{-4}	6.8501×10^{-6}
Length (Å)	250	12
Radius (Å)	63.5	1.9
Axis ratio	1.8	0.1
Power law scale	3.2436×10^{-5}	1.6716×10^{-6}
Power law	2.6	0.01
χ^2	1.1157	

Table S12. Tabulated parameters of the SANS model fit for **Gel-1** sheared at 1 rad/s for 20 minutes.

**Elliptical Cylinder + Power Law**	**Value**	**Error**
Background (cm^{-1})	0.004	
Cylinder scale	2.9551×10^{-4}	1.6791×10^{-5}
Length (Å)	575	135
Radius (Å)	64.6	2.8
Axis ratio	1.8	0.2
Power law scale	3.7240×10^{-5}	3.2524×10^{-6}
Power law	2.6	0.02
χ^2	1.0023	

Table S13. Tabulated parameters of the SANS model fit for **Gel-1** sheared at 10 rad/s for 20 minutes.

**Elliptical Cylinder + Power Law**	**Value**	**Error**
Background (cm^{-1})	0.004	
Cylinder scale	2.6901×10^{-4}	1.6028×10^{-5}
Length (Å)	467	44
Radius (Å)	67.4	2.8
Axis ratio	1.8	0.2
Power law scale	3.7611×10^{-5}	3.1703×10^{-6}
Power law	2.6	0.02
χ^2	1.4830	

Table S14. Tabulated parameters of the SANS model fit for **Gel-1** sheared at 100 rad/s for 20 minutes.

**Sphere + Power Law**	**Value**	**Error**
Background (cm^{-1})	0.004	
Sphere scale	2.0791×10^{-4}	8.4666×10^{-6}
Radius (Å)	108	1.4
Power law scale	3.6696×10^{-5}	2.7813×10^{-6}
Power law	2.7	0.02
χ^2	2.4744	

Table S15. Tabulated parameters of the SANS model fit for **Gel-1** sheared at 1000 rad/s for 20 minutes.

Sphere + Power Law	Value	Error
Background (cm^{-1})	0.004	
Sphere scale	2.0741×10^{-4}	8.4175×10^{-6}
Radius (Å)	107	1.4
Power law scale	3.6963×10^{-5}	2.7545×10^{-6}
Power law	2.7	0.02
χ^2	2.2788	

Table S16. Tabulated parameters of the SANS model fit for **Gel-1** sheared at 2500 rad/s for 20 minutes.

Sphere + Power Law	Value	Error
Background (cm^{-1})	0.004	
Sphere scale	1.11364×10^{-4}	6.8369×10^{-6}
Radius (Å)	111.6	2.2
Power law scale	1.0549×10^{-4}	7.8809×10^{-6}
Power law	2.3	0.02
χ^2	1.507	

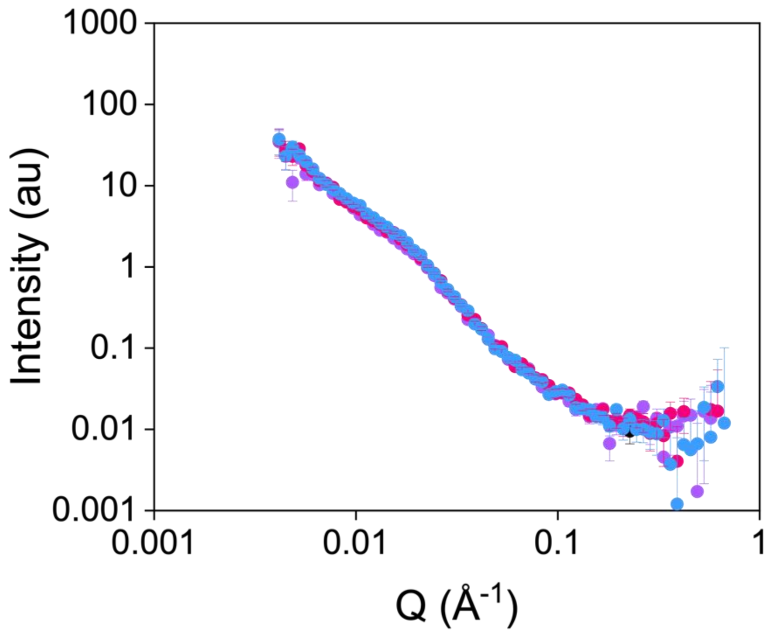

Figure S14. Small-angle neutron scattering patterns for **Gel-1** sheared at 2500 rad/s for 1 second for 1 (purple), 2 (pink), and 3 (blue) cycles.

Table S17. Tabulated parameters of the SANS model fits for **Gel-1** sheared at 2500 rad/s for 1, 2, and 3 cycles.

**Cycle**	**1**	**2**	**3**
Model	Sphere + power law	Sphere + power law	Sphere + power law
Background (cm^{-1})	0.004	0.004	0.004
Sphere scale	$1.11364 \times 10^{-4} \pm 6.8369 \times 10^{-6}$	$1.3727 \times 10^{-4} \pm 7.0353 \times 10^{-6}$	$1.4658 \times 10^{-4} \pm 6.9812 \times 10^{-6}$
Radius (Å)	111.6 ± 2.2	109.2 ± 1.8	110.5 ± 1.7
Power law scale	$1.0549 \times 10^{-4} \pm 7.8809 \times 10^{-6}$	$1.0107 \times 10^{-4} \pm 7.3750 \times 10^{-6}$	$8.1548 \times 10^{-5} \pm 5.9832 \times 10^{-6}$
Power law	2.3 ± 0.02	2.3 ± 0.02	2.4 ± 0.02
χ^2	1.507	1.1928	1.3715

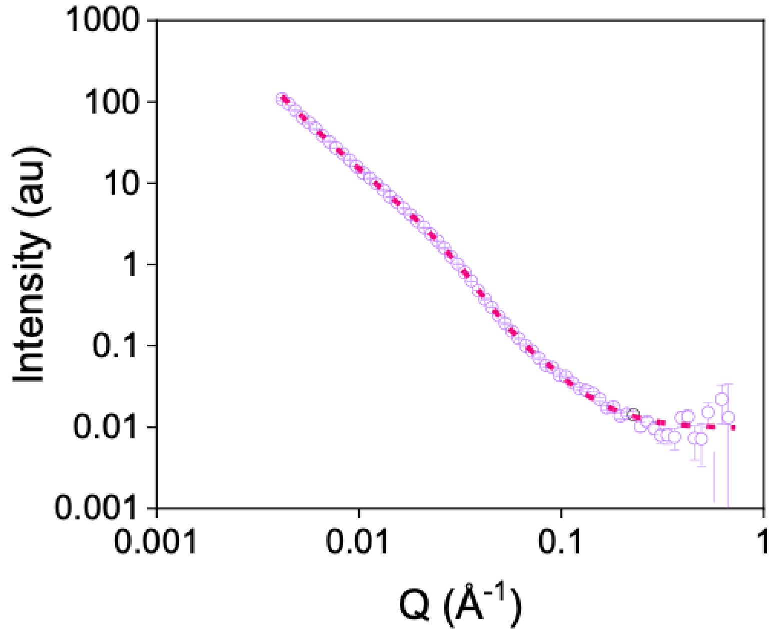

Figure S15. Small-angle neutron scattering patterns for **Gel-1** prepared in a 2 mm cuvette. Open circles show the data and dashed lines represent the fit.

Table S18. Tabulated parameters of the SANS model fit for **Gel-1** prepared in a 2 mm cuvette.

Elliptical Cylinder + Power Law	Value	Error
Background (cm^{-1})	0.01	
Cylinder scale	4.3576×10^{-4}	9.1635×10^{-6}
Length (Å)	541	42
Radius (Å)	54.2	0.9
Axis ratio	1.8	0.05
Power law scale	8.6267×10^{-5}	2.5865×10^{-6}
Power law	2.5	0.006
χ^2	1.5861	

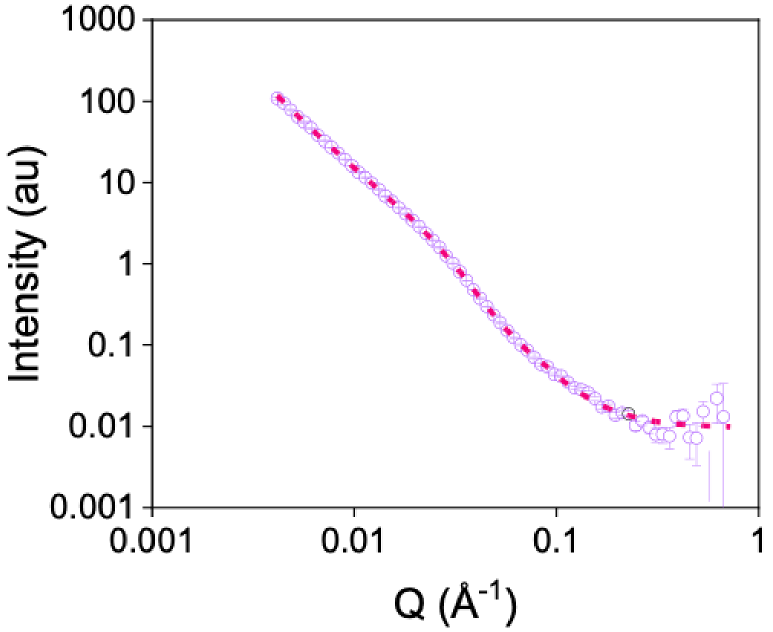

Figure S16. Small-angle neutron scattering patters for **Gel-1** sheared at 1 rad/s using a syringe pump. Open circles show the data and dashed lines represent the fit.

Table S19. Tabulated parameters of the SANS model fit for **Gel-1** sheared at 1 rad/s using a syringe pump.

Elliptical Cylinder + Power Law	Value	Error
Background (cm^{-1})	0.01	
Cylinder scale	4.3576×10^{-4}	9.1635×10^{-6}
Length (Å)	535	35
Radius (Å)	53.7	0.8
Axis ratio	1.8	0.06
Power law scale	8.3675×10^{-5}	2.6545×10^{-6}
Power law	2.5	0.005
χ^2	1.7843	

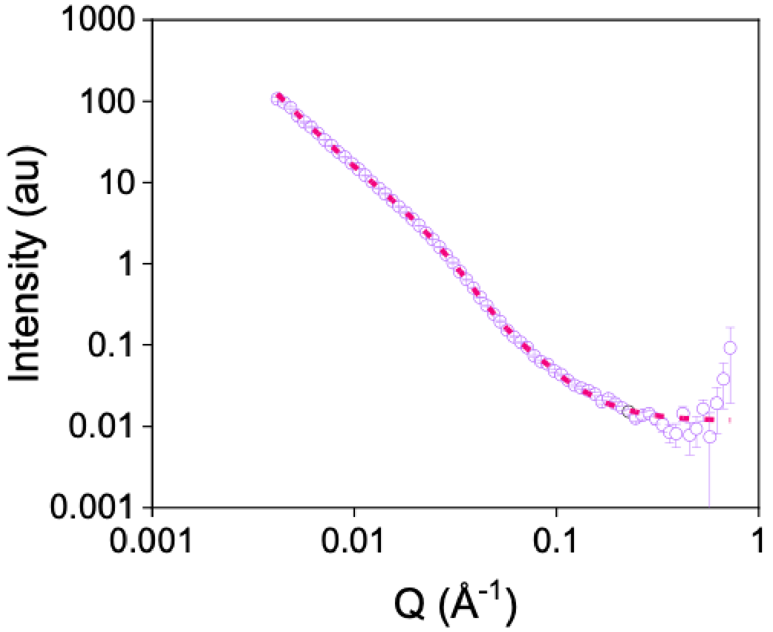

Figure S17. Small-angle neutron scattering patterns for **Gel-1** sheared at 10 rad/s using a syringe pump. Open circles show the data and dashed lines represent the fit.

Table S20. Tabulated parameters of the SANS model fit for **Gel-1** sheared at 10 rad/s using a syringe pump.

**Elliptical Cylinder + Power Law**	**Value**	**Error**
Background (cm^{-1})	0.01	
Cylinder scale	4.4066×10^{-4}	1.0276×10^{-5}
Length (Å)	1000	10
Radius (Å)	54.3	0.9
Axis ratio	1.9	0.06
Power law scale	9.4646×10^{-5}	2.7696×10^{-6}
Power law	2.5	0.005
χ^2	2.1415	

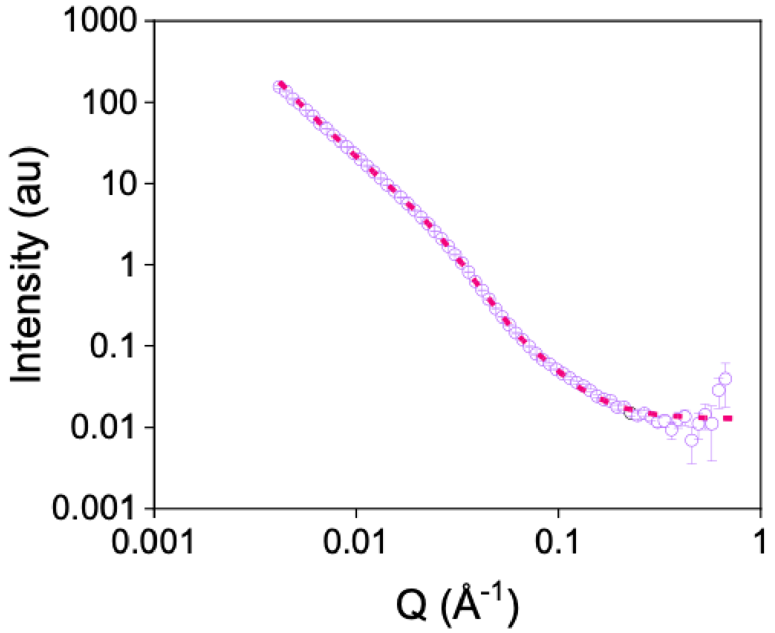

Figure S18. Small-angle neutron scattering patters for **Gel-1** sheared at 100 rad/s using a syringe pump. Open circles show the data and dashed lines represent the fit.

Table S21. Tabulated parameters of the SANS model fit for **Gel-1** sheared at 100 rad/s using a syringe pump.

**Elliptical Cylinder + Power Law**	**Value**	**Error**
Background (cm^{-1})	0.01	
Cylinder scale	6.6441×10^{-4}	1.0978×10^{-5}
Length (Å)	513	25
Radius (Å)	51.9	0.6
Axis ratio	2.1	0.04
Power law scale	7.2330×10^{-5}	2.0633×10^{-6}
Power law	2.6	0.006
χ^2	2.8963	

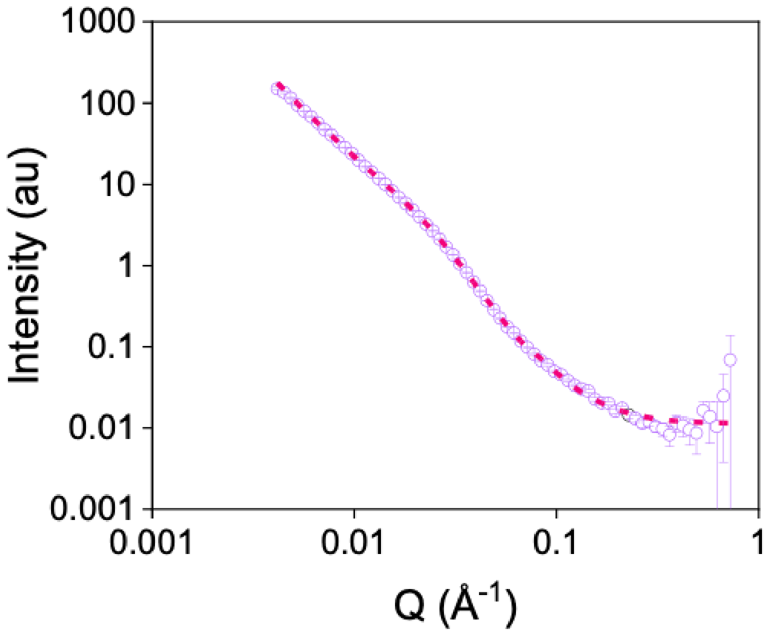

Figure S19. Small-angle neutron scattering patters for **Gel-1** sheared at 1000 rad/s using a syringe pump. Open circles show the data and dashed lines represent the fit.

Table S22. Tabulated parameters of the SANS model fit for **Gel-1** sheared at 1000 rad/s using a syringe pump.

**Elliptical Cylinder + Power Law**	**Value**	**Error**
Background (cm^{-1})	0.01	
Cylinder scale	6.9449×10^{-4}	1.0489×10^{-5}
Length (Å)	547	29
Radius (Å)	53.2	0.6
Axis ratio	2.0	0.04
Power law scale	7.3824×10^{-5}	2.0273×10^{-6}
Power law	2.6	0.006
χ^2	2.5258	

Table S23. Tabulated parameters of the SANS model fit for **Gel-1** sheared at 2500 rad/s using a syringe pump.

**Elliptical Cylinder + Power Law**	**Value**	**Error**
Background (cm^{-1})	0.01	
Cylinder scale	4.2612×10^{-4}	9.1436×10^{-6}
Length (Å)	511	34
Radius (Å)	54.8	0.9
Axis ratio	1.9	0.06
Power law scale	8.3898×10^{-5}	2.4653×10^{-6}
Power law	2.6	0.006
χ^2	2.1387	

3. References

1. E. R. Draper, J. J. Walsh, T. O. McDonald, M. A. Zwijnenburg, P. J. Cameron, A. J. Cowan and D. J. Adams, *J. Mater. Chem. C*, 2014, **2**, 5570–5575.

2 M. C. Nolan, A. M. Fuentes Caparrós, B. Dietrich, M. Barrow, E. R. Cross, M. Bleuel, S. M. King and D. J. Adams, *Soft Matter*, 2017, **13**, 8426–8432.

3 O. Arnold, J. C. Bilheux, J. M. Borreguero, A. Buts, S. I. Campbell, L. Chapon, M. Doucet, N. Draper, R. F. Leal, M. A. Gigg, V. E. Lynch, A. Markvardsen, D. J. Mikkelson, R. L. Mikkelson, R. Miller, K. Palmen, P. Parker, G. Passos, T. G. Perring, P. F. Peterson, S. Ren, M. A. Reuter, A. T. Savici, J. W. Taylor, R. J. Taylor, R. Tolchenov, W. Zhou and J. Zikovsky, *Nucl. Instrum. Methods Phys. Res.*, 2014, **764**, 156-166.

4 https://www.sasview.org/.

5 https://www.ncnr.nist.gov/resources/activation/.
